# *Streptococcus mutans* membrane vesicles inhibit the biofilm formation of *Streptococcus gordonii* and *Streptococcus sanguinis*

**DOI:** 10.1186/s13568-022-01499-3

**Published:** 2022-12-12

**Authors:** Guxin Cui, Pengpeng Li, Ruixue Wu, Huancai Lin

**Affiliations:** 1 Hospital of Stomatology, Guanghua School of Stomatology, Sun Yat-sen University, Guangzhou, China; 2 Guangdong Provincial Key Laboratory of Stomatology, Sun Yat-sen University, Guangzhou, China

**Keywords:** *Streptococcus mutans*, Membrane vesicles, *Streptococcus gordonii*, *Streptococcus sanguinis*, Oral biofilm

## Abstract

*Streptococcus mutans*, whose main virulence factor is glucosyltransferase (Gtf), has a substantial impact on the development of dental caries. *S. mutans* membrane vesicles (MVs), which are rich in Gtfs, have been shown to affect biofilm formation of other microorganisms. *Streptococcus gordonii* and *Streptococcus sanguinis* are initial colonizers of tooth surfaces, which provide attachment sites for subsequent microorganisms and are crucial in the development of oral biofilms. *S. mutans* and *S. gordonii*, as well as *S. mutans* and *S. sanguinis*, have a complex competitive and cooperative relationship, but it is unclear whether *S. mutans* MVs play a role in these interspecific interactions. Therefore, we co-cultured *S. mutans* MVs, having or lacking Gtfs, with *S. gordonii* and *S. sanguinis*. Our results showed that *S. mutans* MVs inhibited biofilm formation of *S. gordonii* and *S. sanguinis* but did not affect their planktonic growth; contrastingly, *S. mutans* Δ*gtfBC* mutant MVs had little effect on both their growth and biofilm formation. Additionally, there were fewer and more dispersed bacteria in the biofilms of the *S. mutans* MV-treated group than that in the control group. Furthermore, the expression levels of the biofilm-related virulence factors *GtfG*, *GtfP*, and *SpxB* in *S. gordonii* and *S. sanguinis* were significantly downregulated in response to *S. mutans* MVs. In conclusion, the results of our study showed that *S. mutans* MVs inhibited biofilm formation of *S. gordonii* and *S. sanguinis*, revealing an important role for MVs in interspecific interactions.

**Supplementary Information:**

The online version contains supplementary material available at 10.1186/s13568-022-01499-3.

## Introduction

Dental caries is a multifactorial disease, affecting 60–90% of children and the majority of adults (Du et al. 2018; Petersen et al. 2005). Biofilms are highly dynamic and structured microbial cell communities that adhere firmly to surfaces and are embedded in self-generated extracellular matrices (Flemming and Wingender 2010). Microbial colonization on tooth surfaces and the formation of cariogenic biofilms are key causes of dental caries (Hara and Zero 2010; Marsh and Zaura 2017; Pitts et al. 2017). Bacterial interactions in biofilms are essential for the development of multispecies microbial communities and for the transition from a healthy to a diseased state of the oral cavity (Hojo et al. 2009; Huang et al. 2011; Marsh and Zaura 2017).

*Streptococcus mutans* plays a crucial role in the development of dental caries (Pitts et al. 2017), given their abilities such as adhesion (Esberg et al. 2012), acid production (Lemos et al. 2005), acid resistance and biofilm formation (Hwang et al. 2014; Quivey et al. 2000). *Streptococcus gordonii* and *Streptococcus sanguinis* are both commensals in the oral cavity that are the initial colonizers of teeth surfaces, and are generally associated with lower levels of cariogenic *S. mutans* and improved dental health (Abranches et al. 2018; Becker et al. 2002; Caufield et al. 2000). There is a complex relationship of competition and cooperation between *S. mutans* and *S. gordonii,* as well as *S. mutans* and *S. sanguinis*. According to previous studies, *S. mutans* inhibits the growth of *S. gordonii* and *S. sanguinis* primarily by releasing bacteriocins, whereas *S. gordonii* and *S. sanguinis* hinder the growth of *S. mutans* mainly through H_2_O_2_ production (Becker et al. 2002; Ge et al. 2008; Kreth et al. 2005, 2008; Wang and Kuramitsu 2005).

Bacterial membrane vesicles (MVs), with a size range of 20–400 nm, carry a variety of cargo molecules, such as nucleic acids, proteins, enzymes, and toxins (Cao and Lin 2021; Toyofuku et al. 2019). *S. mutans* MVs have been successfully isolated and identified from a culture supernatant in 2014 (Liao et al. 2014), providing the foundation for research in this field. Glucosyltransferases (Gtfs) are the major proteins in *S. mutans* MVs; the primary role of these proteins is to utilize sucrose to form extracellular polysaccharides and aid biofilm formation of *S. mutans* (Bowen and Koo 2011; Cao et al. 2020; Rainey et al. 2019). Many oral bacteria, including those that do not synthesize Gtfs, can bind to Gtfs (Vacca-Smith and Bowen 1998). *S. mutans* and *S. mutans* Δ*gtfBC* mutants, as well as early colonizers on the tooth surface such as *Streptococcus mitis*, *Streptococcus oralis*, *S. gordonii*, and *S. sanguinis*, are highly inducible by the complex of MVs, Gtfs, and DNA to form Gtf-dependent biofilms (Senpuku et al. 2019). Our previous study also found that *S. mutans* MVs, which harbor Gtfs, can promote *Candida albicans* biofilm formation (Wu et al. 2020). These observations reveal an important role for *S. mutans* MVs in biofilm formation and interspecies interactions. However, it is unclear whether *S. mutans* MVs play a role in the interspecific interactions between *S. mutans, S. gordonii*, and *S. sanguinis*. Therefore, we questioned whether *S. mutans* MVs that contained or lacked Gtfs affected the growth and biofilm formation of *S. gordonii* and *S. sanguinis*.

In this study, we investigated the effects of *S. mutans* MVs on biofilm formation by *S. gordonii* and *S. sanguinis* by examining the biomass and surface structure of the biofilms. In addition, we analyzed the mechanism by which *S. mutans* MVs affect biofilm development of *S. gordonii* and *S. sanguinis*. Our findings will provide new insights into the interactions between *S. mutans*, *S. gordonii*, *S. sanguinis*, which may be a target for dental caries prevention.

## Materials and methods

### Bacterial strains and culture conditions

*S. mutans* UA159 (ATCC 700610), *S. mutans* UA159 Δ*gtfBC* mutant (Gong et al. 2018; Wu et al. 2020), *S. gordonii* (DL-1), and *S. sanguinis* (ATCC 10556) were grown in a brain heart infusion (BHI; Difco, Detroit, MI, USA) medium at 37 °C under anaerobic conditions (80% N_2_, 10% H_2_, and 10% CO_2_).

### Preparation of MVs

The preparations of *S. mutans* MVs and *S. mutans* Δ*gtfBC* mutant MVs were performed as per a previously described method with a few modifications (Liao et al. 2014). Briefly, the two *S. mutans* strains were incubated in 500 mL of BHI medium at 37 °C for 16 h. After centrifugation at 6000×*g* for 15 min at 4 °C, followed by 10,000×*g* for 15 min at 4 °C, most of the cells in the culture supernatants were removed. After being filtered through 0.22-μm filters (Millipore, MMAS, USA) to remove residual cells, the cell-free culture supernatants were concentrated using a 100 kDa Amicon ultrafiltration system (Millipore, MMAS, USA) at 4000×*g* for 30 min at 4 °C. The collected concentrate was subjected to an initial ultracentrifugation at 100,000×*g* for 70 min at 4 °C; the precipitate was resuspended in sterile phosphate-buffered saline (PBS) and subjected to ultracentrifugation under the same conditions. We then resuspended the precipitates, obtained from the second ultracentrifugation, in 2 mL sterile PBS for subsequent experiments. MV protein concentration was estimated using a BCA Protein Assay Kit (CWBIO, Beijing, China). Finally, the MVs were frozen at − 80 °C at 100 μg/mL until further experimentation.

### Biofilm formation assay by crystal violet staining

Biofilms from *S. gordonii* and *S. sanguinis* were developed in 96-well plates, which were previously coated with artificial saliva at 4 °C for 16 h. *S. gordonii* and *S. sanguinis* were grown anaerobically in BHI broth overnight, and the overnight cultures were inoculated at a ratio of 1:50 into 0.25% BHIS (BHI medium supplemented with 0.25% sucrose) to form the bacterial solutions. Each well contained 100 μL of one of the bacterial solutions and 100 μL of PBS, *S. mutans* MVs, or *S. mutans* Δ*gtfBC* mutant MVs. The MVs were diluted to the desired concentrations with PBS. After 24 h, the biomass of the biofilms was calculated by crystal violet staining. In brief, supernatants and unbound bacteria were removed using three sterile PBS washes, after which 150 μL of absolute methanol was added to fix the biofilms for 15 min. The fixed biofilms were then stained with 0.1% (w/v) crystal violet for 15 min, and the stained areas were checked by gently washing with flowing water until no more dye was evident in the clean wells. Subsequently, the 96-well plates were allowed to dry naturally at about 23–27 °C. Crystal violet was solubilized in 95% ethanol at about 23–27 °C in dark for 30 min. Subsequently, the solubilized crystal violet was transferred to new 96-well plates to measure the absorbance of 95% ethanol solution at 595 nm using a spectrophotometer (Tecan, Reading, Switzerland). Preliminary screening confirmed that the effective concentrations of MVs for *S. gordonii* and *S. sanguinis* were 1 and 0.2 μg/mL, respectively.

### Confocal laser scanning microscopy

Biofilm biomass was measured using confocal laser scanning microscopy (CLSM). The corresponding method was similar to the “Biofilm formation assay by crystal violet staining” method. We performed the experiment in three groups: *S. gordonii* or *S. sanguinis* with PBS; *S. gordonii* or *S. sanguinis* with *S. mutans* MVs; and *S. gordonii* or *S. sanguinis* with *S. mutans* Δ*gtfBC* mutant MVs. *S. gordonii*, and the *S. sanguinis* biofilms were developed on confocal dishes for 24 h, washed thrice with sterile PBS to remove the supernatants and floating cells, stained with 2.5 μM SYTO-9 (Invitrogen Corp., Carlsbad, CA, USA) in dark for 15 min, and observed using CLSM (Olympus, FV3000, Japan). The excitation wavelength of SYTO-9 was 488 nm. CLSM images were collected from three distinct regions of three biological samples. The biomass of *S. gordonii* and *S. sanguinis* were calculated using the COMSTAT image analysis system.

### Scanning electron microscopy

The surface structure of the biofilm was observed using scanning electron microscopy (SEM) (Quanta 400F-FEI, Eindhoven, Netherlands). The biofilms were lightly washed with sterile PBS thrice to remove the supernatants and planktonic cells and then fixed with 2.5% (w/v) glutaraldehyde for ≥ 3 h. The fixed biofilms were then washed with sterile PBS four times for 20 min each time, followed by gradual dehydration for 15 min each with 30, 50, 70, 90 and 100% ethanol. The biofilm samples required an overnight and three dehydration cycles in 70 and 100% ethanol. The biofilms were then soaked in tert-butanol three times for 15 min each, dried overnight by lyophilization, and sputtered with gold. Finally, the biofilms were observed using SEM at 2000×, 5000×, and 10,000× magnification.

### Biofilm eradication assay

Biofilm eradication assays were performed according to a modified protocol (Xi et al. 2019). The overnight cultures of *S. gordonii* or *S. sanguinis* were inoculated at a ratio of 1:100 in 200 µL of 0.25% BHIS in 96-well plates at 37 °C under anaerobic conditions. After 24 h, the medium was discarded from the wells and PBS was used to wash the preformed biofilm to remove planktonic bacteria. Then, 100 μL of PBS, *S. mutans* MVs, or *S. mutans* Δ*gtfBC* mutant MVs was added carefully to the wells. MV concentration was 1 μg/mL or 0.2 μg/mL. Next, 100 μL of 0.25% BHIS was added to each well. The plate was cultured for an additional 24 h. Subsequently, the biofilm biomass of each group was calculated using the crystal violet assay.

### CFU counts

The effects of MVs on planktonic growth of *S. gordonii* or *S. sanguinis* at the concentration of 1 or 0.2 μg/mL were evaluated using CFU counts as described previously but with a few changes (Im et al. 2017). Biofilms were cultured according to “Biofilm formation assay by crystal violet staining”. After 24 h, to measure the colonies in the supernatant from the biofilm, the media of the wells were gently mixed as to avoid disturbing the biofilm and 100 μL of the grown culture was serially diluted from ten-fold to 10^6^-fold with PBS, and 10 μL was pipetted and placed on BHI agar plates at 37 °C anaerobically for 24 h. Finally, the number of colonies on each plate was counted.

### Gene expression

Quantitative reverse transcriptase PCR (qRT-PCR) was used to identify the effect of MVs on the expression levels of genes related to biofilm development. *S. gordonii* and *S. sanguinis* biofilms were grown in 6-well plates with or without MVs, using 0.25% BHIS. After 24 h, biofilms were scraped off into 2 mL Eppendorf tubes and centrifugated at 12,000×*g* for 5 min at 4 °C. After being lysed for ≥2 h with 500 μL of 20 mg/mL lysozyme lysate, the bacterial cells were subjected to proteinase K treatment for at least 30 min. Total RNA was extracted from cell pellets using the miRNeasy Mini Kit (QIAGEN GmbH, Hilden, Germany) according to the manufacturer’s instructions, and RNA concentration and purity (A260/A280) were assessed using a NanoDrop 2000 spectrophotometer (Thermo Fisher Scientific, Pittsburgh, PA, USA). RNA was reverse-transcribed using a PrimeScriptTM RT reagent kit (Takara Bio Inc., Otsu, Japan) according to the manufacturer’s instructions. The target RNA was amplified and quantified using the LightCycler 96 Real-Time System using 2× Super SYBR Green qPCR Master mix (ES Science, Shanghai, China). The primers used in this study are listed in Table 1. Finally, the 2^−ΔΔCT^ method was used to quantify the fold changes in gene expression.

**Table 1:** Primers used in this study

Primer name	Sequences (5′–3′)	References
*S. gordonii 16S rRNA*	Forward: AAGCAACGCGAAGAACCTTA	Zheng et al. (2011)
	Reverse: GTCTCGCTAGAGTGCCCAAC	
*S. sanguinis 16S rRNA*	Forward: AGTTGCCATCATTGAGTTG	Zhou et al. (2016)
	Reverse: GTACCAGCCATTGTAACAC	
*GtfG*	Forward: CTTGAATCAGGTGTGATCTA	Lyu et al. (2021)
	Reverse: GGAGTCAGTTCTTGAAGTTTC	
*GtfP*	Forward: GCCCAAATTCTCAACCGTTAC	Zhu et al. (2018)
	Reverse: ATCTTGCCCTTGACTTGGTAG	
*SpxB*	Forward: GGATGCTTTGGCTGAAGAC	Zheng et al. (2011)
	Reverse: GGACCACCTGAACCTACTG	

### Statistical analysis

Biomass biofilms and CFU counts are expressed as mean ± standard deviation (SD) from at least three independent experiments. Statistical significance was evaluated using one-way ANOVA in GraphPad Prism 9 (GraphPad Software, San Diego, CA, USA). Differences between the control and MV-treated groups were compared using Dunnett's multiple comparison test.* P* < 0.05 was considered statistically significant.

## Results

### *S. mutans *MVs inhibit the biofilm formation of *S. gordonii *and *S. sanguinis*

**Fig. 1:**
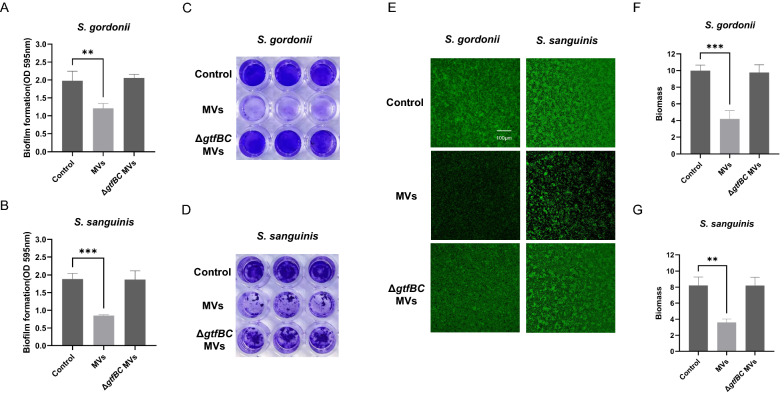
Effects of MVs on the biofilm formation of *S. gordonii* and *S. sanguinis*. **A**, **C** Crystal violet staining applied to *S. gordonii* biofilm. **B**, **D** Crystal violet staining applied to *S. sanguinis* biofilm. **E** CLSM images of *S. gordonii* and *S. sanguinis* biofilms. Images were taken at 20× magnification. **F** The biomass of *S. gordonii* from CLSM images. **G** The biomass of *S. sanguinis* from CLSM images. The data are presented as mean ± SD from three independent experiments. (n = 3, **P < 0.01, ***P < 0.001)

**Fig. 2:**
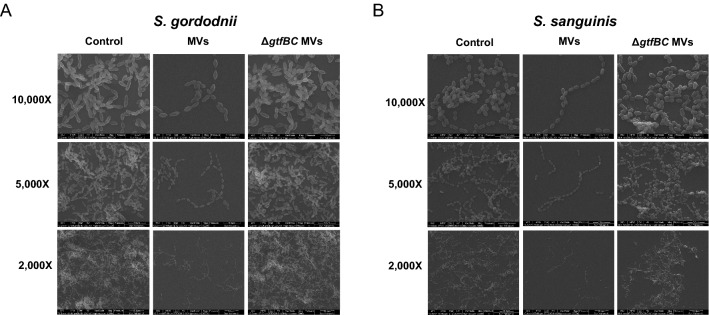
Morphological characteristics of *S. gordonii* and *S. sanguinis* biofilms after treatment with MVs. The bacteria and MVs were co-cultured for 24 h on glass coverslips. Images of *S. gordonii* and *S. sanguinis* biofilms were captured using SEM at 2000×, 5000×, and 10,000× magnifications

Crystal violet staining was used to analyze the effects of *S. mutans* MVs on bacterial biofilm formation. *S. gordonii* and *S. sanguinis* were grown anaerobically in 0.25% BHIS containing a gradient concentration of MVs for 24 h, and biofilm biomass after incubation was quantified by crystal violet staining. The experimental results showed that when the *S. mutans* MVs concentration was approximately 1 μg/mL, the biofilm biomass of *S. gordonii* was significantly reduced compared to that of the control group. For *S. sanguinis*, the concentration of *S. mutans* MVs inhibiting biofilm formation was about 0.2 μg/mL (Additional file 1: Fig. S1). However, no significant differences were observed among *S. mutans* Δ*gtfBC* mutant MV-treated groups (Additional file 1: Fig. S2). We then added the same concentration of *S. mutans* MVs and *S. mutans* Δ*gtfBC* mutant MVs to 0.25% BHIS to develop biofilms of *S. gordonii* and *S. sanguinis*. The crystal violet staining assay results confirmed that *S. mutans* MVs significantly reduced biofilm formation by *S. gordonii* and *S. sanguinis* in contrast to the control group (Fig. 1a–d; *P* < 0.05). Contrastingly, the biofilms of *S. gordonii* and *S. sanguinis* treated with *S. mutans* Δ*gtfBC* mutant MVs were not significantly different from those of the control group (Fig. 1a–d; *P* > 0.05). CLSM also showed that *S. mutans* MVs reduced the aggregation and accumulation of *S. gordonii* and *S. sanguinis* (Fig. 1e). Compared with the control group, the biomass of the *S. mutans* MV-treated group was significantly reduced by approximately 0.6-fold (Fig. 1f, g; *P* < 0.05), whereas there was no significant change in the *S. mutans* Δ*gtfBC* mutant MV-treated group (Fig. 1f, g; *P* > 0.05), which was consistent with subsequent findings. Next, we used SEM to record morphological changes in *S. gordonii* and *S. sanguinis* biofilms. After treatment with *S. mutans* MVs, biofilms were significantly obstructed. The cells of the *S. mutans* MV-treated group were more dispersed than those of the control group under SEM, while the *S. mutans* Δ*gtfBC* mutant MV-treated group showed no significant changes (Fig. 2a, b). Finally, we explored whether *S. mutans* MVs could eradicate the established biofilms of *S. gordonii* and *S. sanguinis*. We applied the MVs to biofilms that had been formed for 24 h and continued to culture for another 24 h. However, the crystal violet assay showed that neither type of MV had a significant scavenging effect on the mature biofilm (Additional file 1: Fig. S3).

### *S. mutans *MVs did not affect the planktonic growth of *S. gordonii* and *S. sanguinis*

**Fig. 3:**
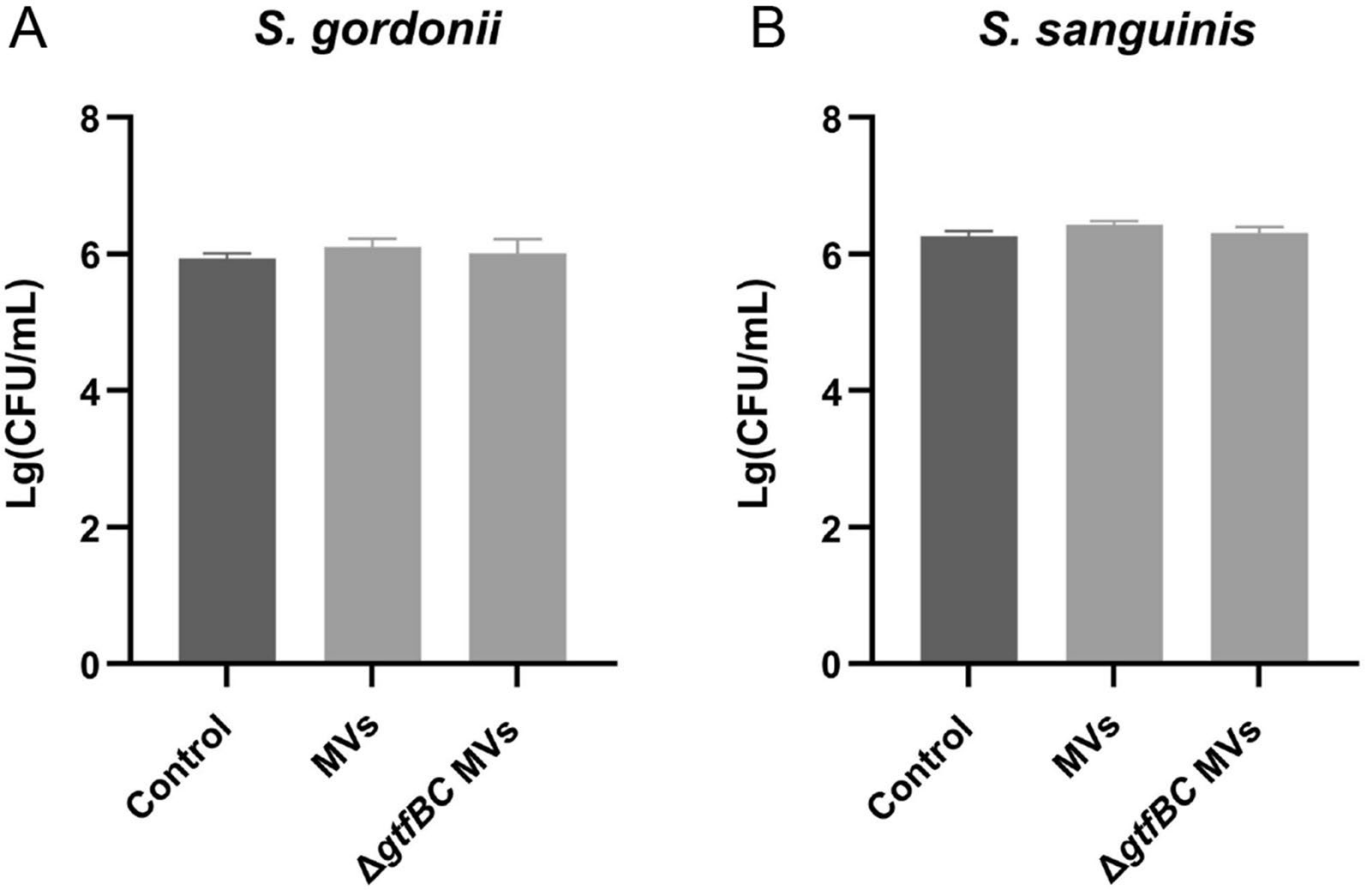
Effects of MVs on the planktonic growth of *S. gordonii* and *S. sanguinis*. **A** CFU counts of *S. gordonii* in the presence or absence of *S. mutans* MVs or *S. mutans* Δ*gtfBC* mutant MVs. **B** CFU counts of *S. sanguinis* in the presence or absence of *S. mutans* MVs or *S. mutans* Δ*gtfBC* mutant MVs. There were no significant differences among the groups. The data are presented as mean ± SD from three independent experiments (n = 3, *P* > 0.05)

Having observed the effects of *S. mutans* MVs on biofilm formation of *S. gordonii* and *S. sanguinis*, we questioned whether *S. mutans* MVs had an effect on the planktonic growth of *S. gordonii* and *S. sanguinis*. We measured the colonies in the supernatant from the biofilm experiments using the CFU count assay. However, no significant differences were found between the *S. mutans* MV-treated group, *S. mutans* Δ*gtfBC* mutant MV-treated group, and control group (Fig. 3a, b;* P* > 0.05). This indicated that *S. mutans* MVs does not affect the growth of *S. gordonii* or *S. sanguinis* under planktonic conditions.

### *S. mutans *MVs inhibited the expression of virulence genes of *S. gordonii* and *S. sanguinis*

**Fig. 4:**
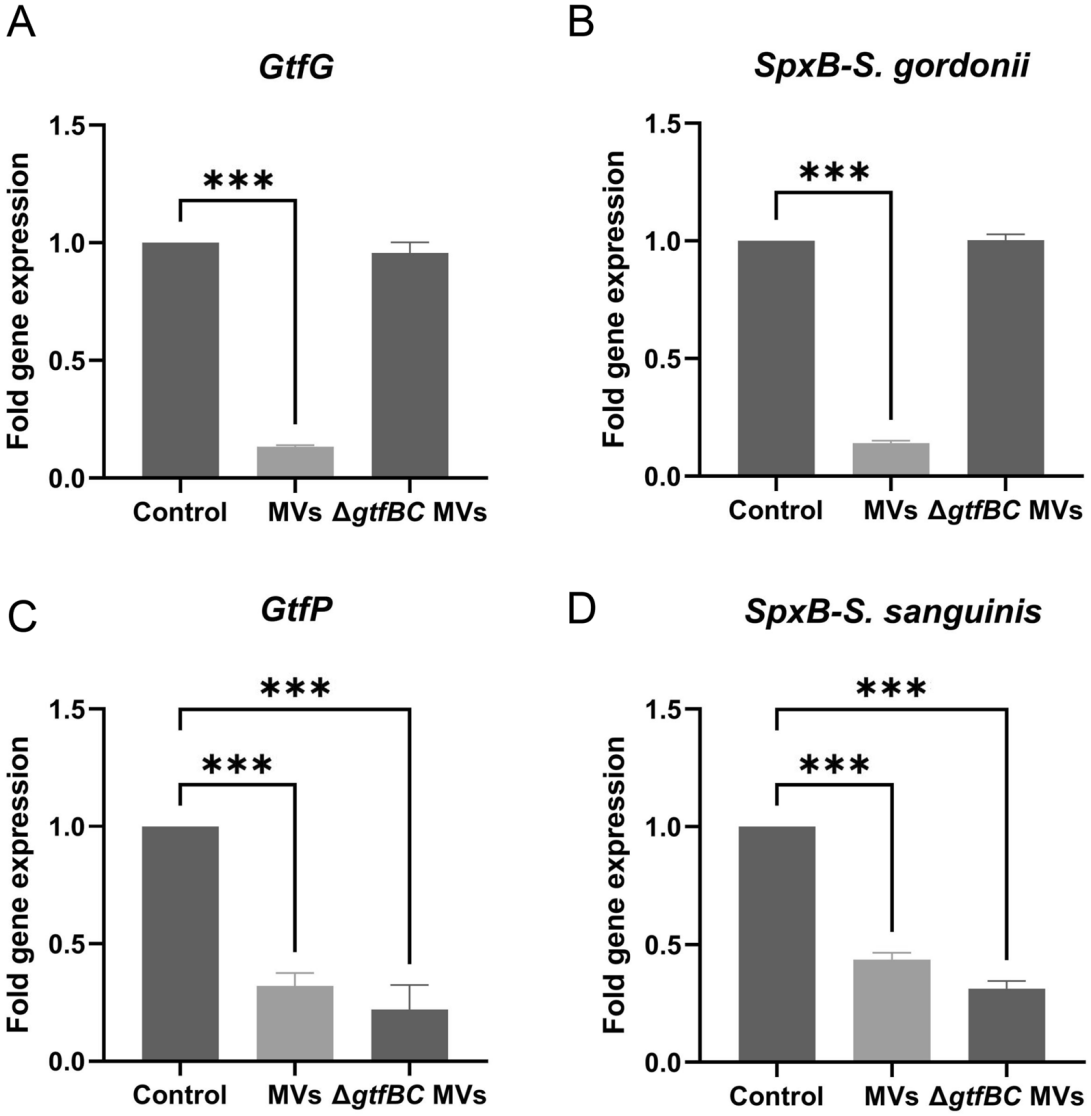
Effects of MVs on virulence genes of *S. gordonii* and *S. sanguinis*. **A** *GtfG*, **B** *SpxB* in *S. gordonii*, **C** *GtfP*, **D** *SpxB* in *S. sanguinis*. The data are presented as mean ± SD from three independent experiments (n = 3, ***P < 0.001)

The next section of this study focused on the expression levels of virulence genes in *S. gordonii* and *S. sanguinis*. qRT-PCR was used to quantify the expression levels of *GtfG*, *GtfP*, and *SpxB* with *S. mutans* MVs treatment. When *S. gordonii* or *S. sanguinis* was incubated in 0.25%BHIS containing 1 μg/mL or 0.2 μg/mL *S. mutans* MVs, the transcription of Gtf genes related to biosynthesis of water-soluble and water-insoluble glucan decreased; this included the *GtfG* gene of *S. gordonii* and the *GtfP* gene of *S. sanguinis *(Vickerman et al. 1997; Xu et al. 2007), which were significantly downregulated 0.9-fold and 0.7-fold, respectively. Contrastingly, in *S. mutans* Δ*gtfBC* mutant MV-treated group, the expression level of *GtfG* did not change significantly, and *GtfP* was downregulated 0.8-fold (Fig. 4a, c; *P* < 0.05). *S. gordonii* and *S. sanguinis* depend on pyruvate oxidase, encoded by *SpxB*, to produce H_2_O_2_ which induces extracellular DNA (eDNA) release and cell aggregation (Itzek et al. 2011; Kreth et al. 2009), thereby promoting biofilm maturation. In our study, the *SpxB* gene was significantly downregulated in *S. gordonii*, 0.9-fold, and *S. sanguinis*, 0.6-fold, after *S. mutans* MVs treatment, whereas in the *S. mutans* Δ*gtfBC* mutant MV-treated group, the expression level of *SpxB* in *S. gordonii* showed little change and was downregulated by approximately 0.7-fold in *S. sanguinis* (Fig. 4b, d; *P* < 0.05).

## Discussion

Given the assumption that vesicles cannot pass through the thick cell walls present in gram-positive bacteria, mycobacteria, and fungi, MVs research in these organisms was disregarded until recently. In 2009, MVs of the gram-positive bacterium, *Staphylococcus aureus*, were extracted from culture supernatants and was shown to enriched with virulence proteins (Lee et al. 2009).Subsequently, MVs secreted by gram-positive bacteria, such as *Mycobacteria *(Prados-Rosales et al. 2011) and *Bacillus subtilis *(Brown et al. 2014) were successively isolated, and the MVs of *S. mutans* were successfully isolated and identified in 2014(Liao et al. 2014). According to recent studies, Gtfs are the major proteins in the MVs of *S. mutans *(Cao et al. 2020; Senpuku et al. 2019). *S. mutans* generates at least three different Gtfs (Bowen and Koo 2011; Rainey et al. 2019), including *GtfB*, *GtfC* and *GtfD*, which are important virulence factors in *S. mutans *(Loesche 1986; Tsumori and Kuramitsu 1997). *GtfB* and *GtfC* are crucial in the metabolism of sucrose; *GtfB* mostly produces water-insoluble glucan, while *GtfC* produces both water-soluble and water-insoluble glucan (Bowen and Koo 2011). We isolated *S. mutans* MVs and *S. mutans* Δ*gtfBC* mutant MVs from culture supernatants, and the morphology of these MVs were observed to be “cup-shaped” and that *S. mutans* MVs had varied size ranges, which was consistent with previous studies on MVs isolated from *S. mutans *(Cao et al. 2020; Liao et al. 2014; Wu et al. 2020).

It is widely recognized that MVs are crucial for intercellular signal transduction and biofilm formation. *S. mutans* MVs affect the development of biofilms in a range of bacteria and fungi (Cao et al. 2020; Liao et al. 2014; Schooling and Beveridge 2006; Senpuku et al. 2019; Wang et al. 2015; Wu et al. 2022, 2020). Antagonism between beneficial commensals (such as *S. gordonii* and *S. sanguinis*) and cariogenic bacteria (such as *S. mutans*) is a major factor affecting the composition and ecology of supragingival biofilms (Huang et al. 2018). Therefore, we questioned whether *S. mutans* MVs that contained or lacked Gtfs had an impact on planktonic growth and biofilm formation of *S. gordonii* and *S. sanguinis*. Our findings demonstrate that *S. gordonii* and *S. sanguinis* biofilm development is inhibited by *S. mutans* MVs without having an impact on the planktonic growth. This is similar to our previous findings on *S. mutans* MVs, which have been shown to affect biofilm formation by *C. albicans* but not their growth (Wu et al. 2020). Similarly, *S. aureus* MVs also affect the biofilm formation of *Acinetobacter baumannii*, *Enterococcus faecium* and *Klebsiella pneumonia*, but not their growth (Im et al. 2017).

However, our findings are in contrast to those of a study conducted by Senpuku et al. showing that *S. mutans* MVs promote the biofilm formation of *S. gordonii* and *S. sanguinis *(Senpuku et al. 2019). This discrepancy may be due to differences in the strains, culture conditions, and detection methods employed. The Senpuku team used *S. gordonii* ATCC10558, whereas we used *S. gordonii* DL-1, which is commonly used in studies of interspecies interactions between *S. mutans* and *S. gordonii *(Huang et al. 2018; Ito et al. 2017; Kreth et al. 2008). In addition, in our biofilm formation experiments, *S. gordonii* and *S. sanguinis* were cultivated in BHI medium containing 0.25% sucrose, and after 24 h, the biomass of the biofilms was observed and measured by crystal violet staining and CLSM. Unlike our approach, Senpuku et al. developed biofilms in TSB medium and quantified the biofilm biomass by safranin staining after 16 h of incubation. Growth medium is one of the key factors affecting MVs production and contents (Klimentova and Stulik 2015). Changes in the composition of the growth medium altered the protein content and immunogenicity of *Neisseria meningitidis* vesicles (Tsolakos et al. 2010). For *Francisella tularensis*, the type of medium had some influence on the resulting bacterial phenotype (Hazlett et al. 2008). This suggests that the type of medium used may influence the effect MVs have on biofilm formation of *S. gordonii* and *S. sanguinis.* Another important difference was the extraction time of the MVs. We isolated MVs from the culture supernatants when *S. mutans* grew for 16 h, whereas the Senpuku team isolated MVs after 24 h. Liao et al. showed that *S. mutans* MVs from early exponential phase cultures contained 2.82-fold more eDNA than those prepared from overnight cultures (Liao et al. 2014). Tashiro et al. showed that vesicles secreted by *Pseudomonas aeruginosa* during the exponential and stationary phases exhibit distinct physicochemical properties; along with the growth transition, the characteristics of vesicles are changed, allowing a greater level of interaction with bacteria (Tashiro et al. 2010). *Francisella novicida* produces more vesicles in the early stationary phase than in the mid-logarithmic phase, and the protein profiles are different as well (McCaig et al. 2013). However, it is not yet known whether there are other differences in the MVs produced by *S. mutans* at the different growth stages, indicating a potential area for future research.

qRT-PCR results showed that the expression levels of *GtfG* in *S. gordonii* and *GtfP* in *S. sanguinis* were significantly downregulated in response to *S. mutans* MVs. *GtfG* and *GtfP* are important virulence factors in *S. gordonii* and *S. sanguinis*. *GtfG*, produced by *S. gordonii*, can synthesize both water-soluble and water-insoluble glucans and regulate the adhesion of *S. gordonii *(Vickerman et al. 1997). *S. sanguinis* has two Gtf genes, *GtfA* and *GtfP *(Xu et al. 2007), of which *GtfP* is the only one that produces glucans. When the *GtfP* is deleted, less water-soluble and water-insoluble glucan are produced, leading to a reduction in biofilm formation (Liu et al. 2017; Yoshida et al. 2014; Zhu et al. 2017). The expression levels of *SpxB* in *S. gordonii* and *S. sanguinis* were also significantly downregulated in response to *S. mutans* MVs. The H_2_O_2_ produced by *SpxB* can inhibit the growth of *S. mutans* and cause the release of eDNA, which is crucial for bacterial adhesion and aggregation during the initial stage of biofilm formation (Das et al. 2010, 2013; Kreth et al. 2008). qRT-PCR results also showed that under the treatment of *S. mutans* Δ*gtfBC* mutant MVs, the expression levels of *GtfG* and *SpxB* in *S. gordonii* did not change significantly compared with the control group, whereas the expression level of these genes in *S. sanguinis* decreased more significantly than those of *S. mutans* MV-treated group. Downregulation of *GtfP* and *SpxB* in *S. sanguinis* has also been observed in previous studies during simultaneous colonization with *S. mutans* under biofilm conditions (Lozano et al. 2019). This implies that *S. mutans* MVs may affect certain bacterial species differently. Gtfs may not be solely responsible for the reduced biofilm formation in *S. gordonii* and *S. sanguinis*. It is likely that other components of MVs are also involved; however, the exact mechanism is unknown. In general, the results indicate that one of the reasons for the decreased biofilm formation of *S. gordonii* and *S. sanguinis* when treated with *S. mutans* MVs may be the decrease in water-soluble and water-insoluble glucan. Furthermore, the reduction of eDNA release, caused by a decrease in H_2_O_2_ production, can cause a corresponding reduction in the matrix of the biofilm and make it less stable. However, since *S. mutans* MVs also contain significant amounts of Gtfs, which work with *GtfG* and *GtfP* to synthesize water-soluble and water-insoluble glucans using sucrose in the culture medium, we were unable to ascertain the amount of glucan reduction produced by *GtfP* and *GtfG*.

When we measured biofilm biomass using crystal violet staining, we found that biofilms in the *S. mutans* MV-treated group were more susceptible to shedding during PBS washing than the other groups, and this observation was especially prominent in *S. gordonii* biofilms. Therefore, we examined the expression levels of several adhesion-related genes of *S. gordonii*, including *AbpA*, *AbpB* and *ScaA* (Additional file 1: Fig. S4). *AbpA* and *AbpB* promote the binding of *S. gordonii* to the acquired pellicle, thus contributing to bacterial colonization and biofilm formation; this process becomes difficult for mutant strains lacking *AbpA *(Rogers et al. 2001; Tanzer et al. 2003). *ScaA* regulates the co-aggregation of *S. gordonii* cells (Zheng et al. 2012). Interestingly, we found that the expression levels of all these genes were significantly downregulated in response to *S. mutans* MVs. The downregulation of expression levels of these genes may help explain the reduced cell aggregation and biofilm formation observed in the *S. mutans* MV-treated group in our study. Taken together with the results of the biofilm eradication experiments, we hypothesized that *S. mutans* MVs may play an inhibitory role in the early stages of biofilm formation.

We successfully isolated *S. mutans* MVs and *S. mutans* Δ*gtfBC* mutant MVs under planktonic condition and applied them to both *S. gordonii* and *S. sanguinis* at the same concentration. We found that *S. mutans* MVs inhibited biofilm formation of *S. gordonii* and *S. sanguinis* but did not affect their planktonic growth. However, we experienced some unavoidable limitations across this study. Although the BCA assay is commonly used method to quantify MVs (Aytar Celik et al. 2022; Bitto et al. 2021), using this method to unify the concentration of two kinds of MVs solutions may have some influence on the experimental results because the protein content of the single MV in *S. mutans* and *S. mutans* Δ*gtfBC* mutant may be different. Furthermore, both in vitro and in vivo interactions of *S. mutans* MVs with mixed biofilms of *S. gordonii* and *S. sanguinis* is unclear. The effects of *S. mutans* MVs on species composition, spatial structure, and cariogenicity of mixed-species biofilms in vitro and in vivo are also unknown. In the future, it may be possible to isolate *S. gordonii* and *S. sanguinis* MVs and observe their influence on the growth and biofilm formation of *S. mutans*. This will undoubtedly provide deeper insights into the complicated interspecific interaction mechanism between *S. mutans* and *S. gordonii*, as well as *S. mutans* and *S. sanguinis*, from the perspective of MVs that will benefit our understanding of the functions of MVs.

## Supplementary Information

**Additional file 1. Figure 1. **Effective concentration screening of *S. mutans* MVs for *S. gordonii *and *S. sanguinis*. The biomass of biofilms was calculated by crystal violet staining. The data are presented as mean ± SD from three independent experiments (n = 3, ***P <  0.001). **Figure 2.** Effects of Δ*gtfBC* MVs on the biofilm formation of *S. gordonii *and *S. sanguinis*. The biomass of biofilms was calculated by crystal violet staining. The data are presented as mean ± SD from three independent experiments (n = 3, *P > *0.05). **Figure 3. **Effects of MVs on the mature biofilm of *S. gordonii* and *S. sanguinis*. The biomass of biofilms was calculated by crystal violet staining. The data are presented as mean ± SD from three independent experiments (n = 3, *P* > 0.05). **Figure 4.** Effects of MVs on the expression levels of adhesion genes of *S. gordonii*. The data are presented as mean ± SD from three independent experiments (n = 3, ***P < 0.001).

## Data Availability

The datasets generated and analyzed during the current study are available from the corresponding author on reasonable request.

## Abbreviations

*S. mutans*: *Streptococcus mutans*
MVs: Membrane vesicles
*S. gordonii*: *Streptococcus gordonii*
*S. sanguinis*: *Streptococcus sanguinis*
BHI: Brain heart infusion
CLSM: Confocal laser scanning microscopy
SEM: Scanning electron microscopy
qRT-PCR: Quantitative reverse transcriptase PCR

## References

[CR1] Abranches J, Zeng L, Kajfasz JK, Palmer SR, Chakraborty B, Wen ZT, Richards VP, Brady LJ, Lemos JA (2018). Biology of oral streptococci. Microbiol Spectr.

[CR2] Aytar Celik P, Derkus B, Erdogan K, Barut D, Blaise Manga E, Yildirim Y, Pecha S, Cabuk A (2022). Bacterial membrane vesicle functions, laboratory methods, and applications. Biotechnol Adv.

[CR3] Becker MR, Paster BJ, Leys EJ, Moeschberger ML, Kenyon SG, Galvin JL, Boches SK, Dewhirst FE, Griffen AL (2002). Molecular analysis of bacterial species associated with childhood caries. J Clin Microbiol.

[CR4] Bitto NJ, Zavan L, Johnston EL, Stinear TP, Hill AF, Kaparakis-Liaskos M (2021). Considerations for the analysis of bacterial membrane vesicles: methods of vesicle production and quantification can influence biological and experimental outcomes. Microbiol Spectrum.

[CR5] Bowen WH, Koo H (2011). Biology of *Streptococcus mutans*-derived glucosyltransferases: role in extracellular matrix formation of cariogenic biofilms. Caries Res.

[CR6] Brown L, Kessler A, Cabezas-Sanchez P, Luque-Garcia JL, Casadevall A (2014). Extracellular vesicles produced by the Gram-positive bacterium *Bacillus subtilis* are disrupted by the lipopeptide surfactin. Mol Microbiol.

[CR7] Cao Y, Lin H (2021). Characterization and function of membrane vesicles in Gram-positive bacteria. Appl Microbiol Biotechnol.

[CR8] Cao Y, Zhou Y, Chen D, Wu R, Guo L, Lin H (2020). Proteomic and metabolic characterization of membrane vesicles derived from *Streptococcus mutans* at different pH values. Appl Microbiol Biotechnol.

[CR9] Caufield PW, Dasanayake AP, Li Y, Pan Y, Hsu J, Hardin JM (2000). Natural history of *Streptococcus sanguinis* in the oral cavity of infants: evidence for a discrete window of infectivity. Infect Immun.

[CR10] Das T, Sharma PK, Busscher HJ, van der Mei HC, Krom BP (2010). Role of extracellular DNA in initial bacterial adhesion and surface aggregation. Appl Environ Microbiol.

[CR11] Das T, Sehar S, Manefield M (2013). The roles of extracellular DNA in the structural integrity of extracellular polymeric substance and bacterial biofilm development. Environ Microbiol Rep.

[CR12] Du MQ, Li Z, Jiang H, Wang X, Feng XP, Hu DY, Lin HC, Wang B, Si Y, Wang CX, Zheng SG, Liu XN, Rong WS, Wang WJ, Tai BJ (2018). Dental caries status and its associated factors among 3- to 5-year-old children in china: a national survey. Chin J Dental Res.

[CR13] Esberg A, Lofgren-Burstrom A, Ohman U, Stromberg N (2012). Host and bacterial phenotype variation in adhesion of *Streptococcus mutans* to matched human hosts. Infect Immun.

[CR14] Flemming HC, Wingender J (2010). The biofilm matrix. Nat Rev Microbiol.

[CR15] Ge Y, Caufield PW, Fisch GS, Li Y (2008). *Streptococcus mutans* and *Streptococcus sanguinis* colonization correlated with caries experience in children. Caries Res.

[CR16] Gong T, Tang BY, Zhou XD, Zeng JM, Lu M, Guo XX, Peng X, Lei L, Gong B, Li YQ (2018). Genome editing in *Streptococcus mutans* through self-targeting CRISPR arrays. Mol Oral Microbiol.

[CR17] Hara AT, Zero DT (2010). The caries environment: saliva, pellicle, diet, and hard tissue ultrastructure. Dent Clin North Am.

[CR18] Hazlett KRO, Caldon SD, McArthur DG, Cirillo KA, Kirimanjeswara GS, Magguilli ML, Malik M, Shah A, Broderick S, Golovliov I, Metzger DW, Rajan K, Sellati TJ, Loegering DJ (2008). Adaptation of *Francisella tularensis* to the mammalian environment is governed by cues which can be mimicked in vitro. Infect Immun.

[CR19] Hojo K, Nagaoka S, Ohshima T, Maeda N (2009). Bacterial interactions in dental biofilm development. J Dent Res.

[CR20] Huang R, Li M, Gregory RL (2011). Bacterial interactions in dental biofilm. Virulence.

[CR21] Huang XL, Browngardt CM, Jiang M, Ahn SJ, Burne RA, Nascimento MM (2018). Diversity in antagonistic interactions between commensal oral streptococci and *Streptococcus mutans*. Caries Res.

[CR22] Hwang G, Klein MI, Koo H (2014). Analysis of the mechanical stability and surface detachment of mature *Streptococcus mutans* biofilms by applying a range of external shear forces. Biofouling.

[CR23] Im H, Lee S, Soper SA, Mitchell RJ (2017). *Staphylococcus aureus* extracellular vesicles (EVs): surface-binding antagonists of biofilm formation. Mol Biosyst.

[CR24] Ito T, Ichinosawa T, Shimizu T (2017). Streptococcal adhesin SspA/B analogue peptide inhibits adherence and impacts biofilm formation of *Streptococcus mutans*. PLoS ONE.

[CR25] Itzek A, Zheng L, Chen Z, Merritt J, Kreth J (2011). Hydrogen peroxide-dependent DNA release and transfer of antibiotic resistance genes in *Streptococcus gordonii*. J Bacteriol.

[CR26] Klimentova J, Stulik J (2015). Methods of isolation and purification of outer membrane vesicles from gram-negative bacteria. Microbiol Res.

[CR27] Kreth J, Merritt J, Shi WY, Qi FX (2005). Competition and coexistence between *Streptococcus mutans* and *Streptococcus sanguinis* in the dental biofilm. J Bacteriol.

[CR28] Kreth J, Zhang Y, Herzberg MC (2008). Streptococcal antagonism in oral biofilms: *Streptococcus sanguinis* and *Streptococcus gordonii* interference with *Streptococcus mutans*. J Bacteriol.

[CR29] Kreth J, Vu H, Zhang Y, Herzberg MC (2009). Characterization of hydrogen peroxide-induced DNA release by *Streptococcus sanguinis* and *Streptococcus gordonii*. J Bacteriol.

[CR30] Lee EY, Choi DY, Kim DK, Kim JW, Park JO, Kim S, Kim SH, Desiderio DM, Kim YK, Kim KP, Gho YS (2009). Gram-positive bacteria produce membrane vesicles: proteomics-based characterization of *Staphylococcus aureus*-derived membrane vesicles. Proteomics.

[CR31] Lemos JA, Abranches J, Burne RA (2005). Responses of cariogenic streptococci to environmental stresses. Curr Issues Mol Biol.

[CR32] Liao SM, Klein MI, Heim KP, Fan YW, Bitoun JP, Ahn SJ, Burne RA, Koo H, Brady LJ, Wen ZZT (2014). *Streptococcus mutans* extracellular DNA is upregulated during growth in biofilms, actively released via membrane vesicles, and influenced by components of the protein secretion machinery. J Bacteriol.

[CR33] Liu J, Stone VN, Ge X, Tang M, Elrami F, Xu P (2017). TetR family regulator brpT modulates biofilm formation in *Streptococcus sanguinis*. PLoS ONE.

[CR34] Loesche WJ (1986). Role of *Streptococcus mutans* in human dental decay. Microbiol Rev.

[CR35] Lozano CP, Diaz-Garrido N, Kreth J, Giacaman RA (2019). *Streptococcus mutans* and *Streptococcus sanguinis* expression of competition-related genes, under sucrose. Caries Res.

[CR36] Lyu X, Wang L, Shui Y, Jiang Q, Chen L, Yang W, He X, Zeng J, Li Y (2021). Ursolic acid inhibits multi-species biofilms developed by *Streptococcus mutans*, *Streptococcus sanguinis*, and *Streptococcus gordonii*. Arch Oral Biol.

[CR37] Marsh PD, Zaura E (2017). Dental biofilm: ecological interactions in health and disease. J Clin Periodontol.

[CR38] McCaig WD, Koller A, Thanassi DG (2013). Production of outer membrane vesicles and outer membrane tubes by *Francisella novicida*. J Bacteriol.

[CR39] Petersen PE, Bourgeois D, Ogawa H, Estupinan-Day S, Ndiaye C (2005). The global burden of oral diseases and risks to oral health. Bull World Health Organ.

[CR40] Pitts NB, Zero DT, Marsh PD, Ekstrand K, Weintraub JA, Ramos-Gomez F, Tagami J, Twetman S, Tsakos G, Ismail A (2017). Dental caries. Nat Rev Dis Primers.

[CR41] Prados-Rosales R, Baena A, Martinez LR, Luque-Garcia J, Kalscheuer R, Veeraraghavan U, Camara C, Nosanchuk JD, Besra GS, Chen B, Jimenez J, Glatman-Freedman A, Jacobs WR, Porcelli SA, Casadevall A (2011). Mycobacteria release active membrane vesicles that modulate immune responses in a TLR2-dependent manner in mice. J Clin Invest.

[CR42] Quivey RG, Kuhnert WL, Hahn K (2000). Adaptation of oral streptococci to low pH. Adv Microb Physiol.

[CR43] Rainey K, Michalek SM, Wen ZT, Wu H (2019). Glycosyltransferase-mediated biofilm matrix dynamics and virulence of *Streptococcus mutans*. Appl Environ Microbiol.

[CR44] Rogers JD, Palmer RJ, Kolenbrander PE, Scannapieco FA (2001). Role of *Streptococcus gordonii* amylase-binding protein A in adhesion to hydroxyapatite, starch metabolism, and biofilm formation. Infect Immun.

[CR45] Schooling SR, Beveridge TJ (2006). Membrane vesicles: an overlooked component of the matrices of biofilms. J Bacteriol.

[CR46] Senpuku H, Nakamura T, Iwabuchi Y, Hirayama S, Nakao R, Ohnishi M (2019). Effects of complex DNA and MVs with GTF extracted from *Streptococcus mutans* on the oral biofilm. Molecules.

[CR47] Tanzer JM, Grant L, Thompson A, Li L, Rogers JD, Haase EM, Scannapieco FA (2003). Amylase-binding proteins A (AbpA) and B (AbpB) differentially affect colonization of rats' teeth by *Streptococcus gordonii*. Microbiology (reading).

[CR48] Tashiro Y, Ichikawa S, Shimizu M, Toyofuku M, Takaya N, Nakajima-Kambe T, Uchiyama H, Nomura N (2010). Variation of physiochemical properties and cell association activity of membrane vesicles with growth phase in *Pseudomonas aeruginosa*. Appl Environ Microbiol.

[CR49] Toyofuku M, Nomura N, Eberl L (2019). Types and origins of bacterial membrane vesicles. Nat Rev Microbiol.

[CR50] Tsolakos N, Lie K, Bolstad K, Maslen S, Kristiansen PA, Hoiby EA, Wallington A, Vipond C, Skehel M, Tang CM, Feavers IM, Wedege E, Wheeler JX (2010). Characterization of meningococcal serogroup B outer membrane vesicle vaccines from strain 44/76 after growth in different media. Vaccine.

[CR51] Tsumori H, Kuramitsu H (1997). The role of the *Streptococcus mutans* glucosyltransferases in the sucrose-dependent attachment to smooth surfaces: essential role of the GtfC enzyme. Oral Microbiol Immunol.

[CR52] Vacca-Smith AM, Bowen WH (1998). Binding properties of streptococcal glucosyltransferases for hydroxyapatite, saliva-coated hydroxyapatite, and bacterial surfaces. Arch Oral Biol.

[CR53] Vickerman MM, Sulavik MC, Nowak JD, Gardner NM, Jones GW, Clewell DB (1997). Nucleotide sequence analysis of the *Streptococcus gordonii* glucosyltransferase gene, gtfG. DNA Seq.

[CR54] Wang BY, Kuramitsu HK (2005). Interactions between oral bacteria: Inhibition of *Streptococcus mutans* bacteriocin production by *Streptococcus gordonii*. Appl Environ Microb.

[CR55] Wang WD, Chanda W, Zhong MT (2015). The relationship between biofilm and outer membrane vesicles: a novel therapy overview. Fems Microbiol Lett.

[CR56] Wu R, Tao Y, Cao Y, Zhou Y, Lin H (2020). *Streptococcus mutans* membrane vesicles harboring glucosyltransferases augment *Candida albicans* biofilm development. Front Microbiol.

[CR57] Wu R, Cui G, Cao Y, Zhao W, Lin H (2022). *Streptococcus mutans* membrane vesicles enhance *Candida albicans* pathogenicity and carbohydrate metabolism. Front Cell Infect Microbiol.

[CR58] Xi Y, Wang Y, Gao J, Xiao Y, Du J (2019). Dual corona vesicles with intrinsic antibacterial and enhanced antibiotic delivery capabilities for effective treatment of biofilm-induced periodontitis. ACS Nano.

[CR59] Xu P, Alves JM, Kitten T, Brown A, Chen Z, Ozaki LS, Manque P, Ge X, Serrano MG, Puiu D, Hendricks S, Wang Y, Chaplin MD, Akan D, Paik S, Peterson DL, Macrina FL, Buck GA (2007). Genome of the opportunistic pathogen *Streptococcus sanguinis*. J Bacteriol.

[CR60] Yoshida Y, Konno H, Nagano K, Abiko Y, Nakamura Y, Tanaka Y, Yoshimura F (2014). The influence of a glucosyltransferase, encoded by gtfP, on biofilm formation by *Streptococcus sanguinis* in a dual-species model. APMIS.

[CR61] Zheng L, Itzek A, Chen Z, Kreth J (2011). Environmental influences on competitive hydrogen peroxide production in *Streptococcus gordonii*. Appl Environ Microbiol.

[CR62] Zheng L, Chen Z, Itzek A, Herzberg MC, Kreth J (2012). CcpA regulates biofilm formation and competence in *Streptococcus gordonii*. Mol Oral Microbiol.

[CR63] Zhou Y, Yang J, Zhang L, Zhou X, Cisar JO, Palmer RJ (2016). Differential utilization of basic proline-rich glycoproteins during growth of oral bacteria in saliva. Appl Environ Microbiol.

[CR64] Zhu B, Ge X, Stone V, Kong X, El-Rami F, Liu Y, Kitten T, Xu P (2017). ciaR impacts biofilm formation by regulating an arginine biosynthesis pathway in *Streptococcus sanguinis* SK36. Sci Rep.

[CR65] Zhu B, Song L, Kong X, Macleod LC, Xu P (2018). A novel regulator modulates glucan production, cell aggregation and biofilm formation in *Streptococcus sanguinis* SK36. Front Microbiol.

