# Supplementary material for: Streptococcus mutans membrane vesicles inhibit the biofilm formation of Streptococcus gordonii and Streptococcus sanguinis
Source: AMB Express. 2022 Dec 12;12:154. doi: 10.1186/s13568-022-01499-3 (PMC9743899; doi:10.1186/s13568-022-01499-3)
Supplement: Supplementary file 1 — Additional file 1. Figure 1. Effective concentration screening of S. mutans MVs for S. gordonii and S. sanguinis. The biomass of biofilms was calculated by crystal violet staining. The data are presented as mean ± SD from three independent experiments (n = 3, ***P < 0.001). Figure 2. Effects of ΔgtfBC MVs on the biofilm formation of S. gordonii and S. sanguinis. The biomass of biofilms was calculated by crystal violet staining. The data are presented as mean ± SD from three independent experiments (n = 3, P > 0.05). Figure 3. Effects of MVs on the mature biofilm of S. gordonii and S. sanguinis. The biomass of biofilms was calculated by crystal violet staining. The data are presented as mean ± SD from three independent experiments (n = 3, P > 0.05). Figure 4. Effects of MVs on the expression levels of adhesion genes of S. gordonii. The data are presented as mean ± SD from three independent experiments (n = 3, ***P < 0.001). [file 13568_2022_1499_MOESM1_ESM.docx]

**Supplemental Materials**

| A  **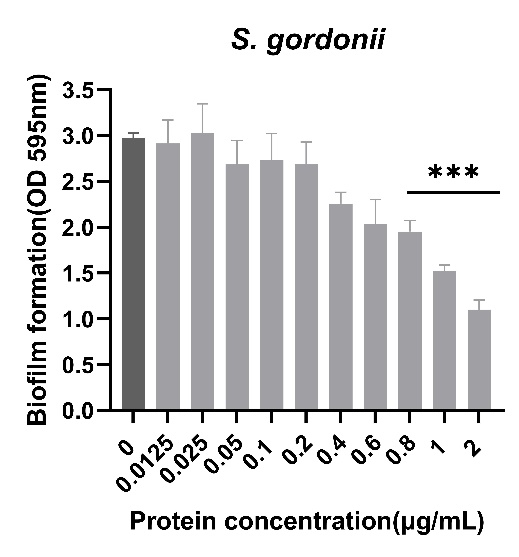** | B  **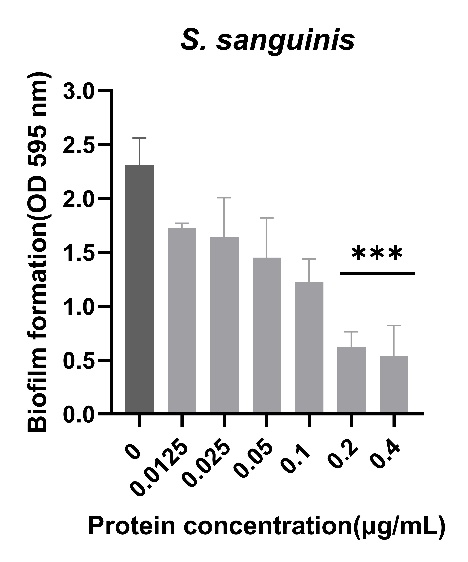** |
| --- | --- |

**Figure 1. Effective concentration screening of *S. mutans* MVs for *S. gordonii* and *S. sanguinis*.** The biomass of biofilms was calculated by crystal violet staining. The data are presented as mean ± SD from three independent experiments (n = 3, ∗∗∗*P* < 0.001).

| A  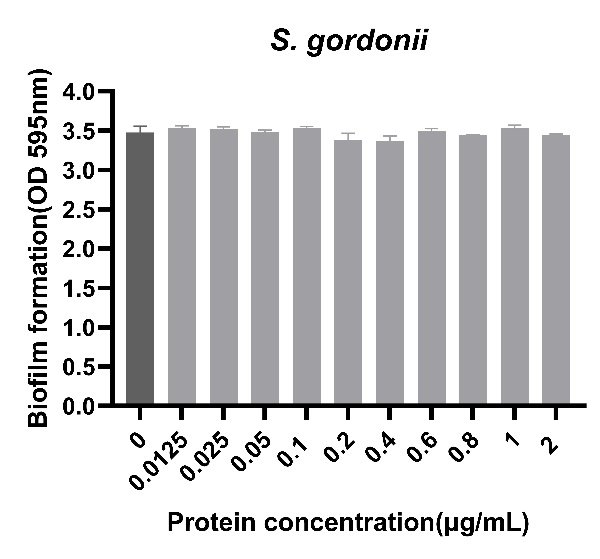 | B  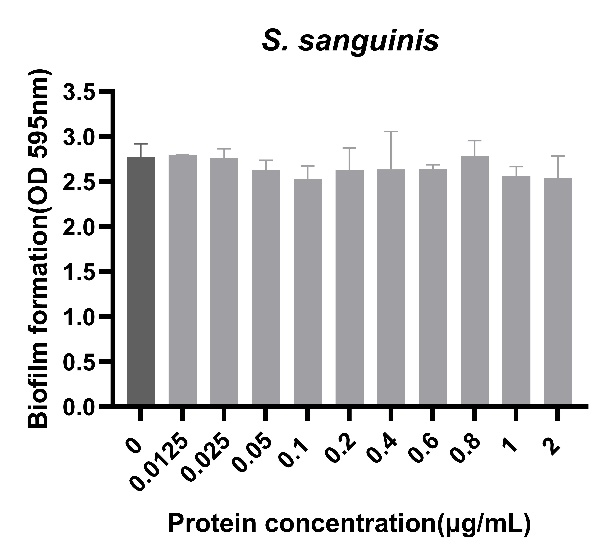 |
| --- | --- |

**Figure 2. Effects of Δ*gtfBC* MVs on the biofilm formation of *S. gordonii*** **and *S. sanguinis*.** The biomass of biofilms was calculated by crystal violet staining. The data are presented as mean ± SD from three independent experiments (n = 3, *P*＞0.05).

| A  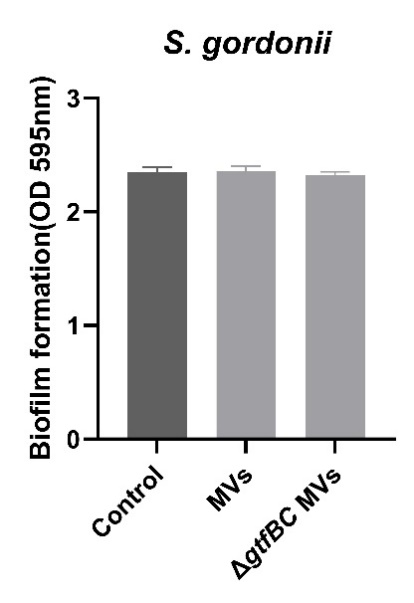 | B  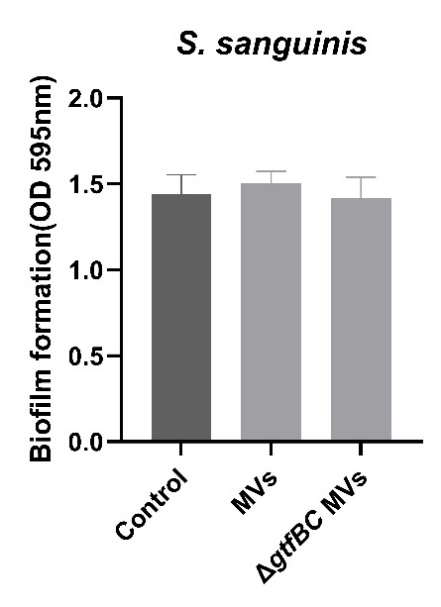 |
| --- | --- |

**Figure 3. Effects of MVs on the mature biofilm of *S. gordonii* and *S. sanguinis*.** The biomass of biofilms was calculated by crystal violet staining. The data are presented as mean ± SD from three independent experiments (n = 3, *P*＞0.05).

| A  **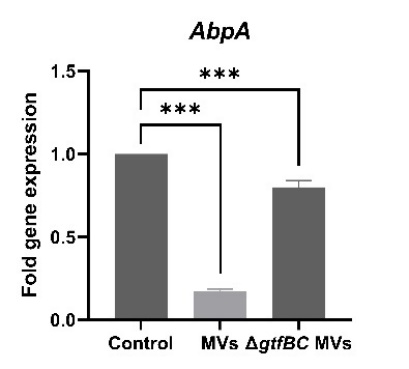** | B  **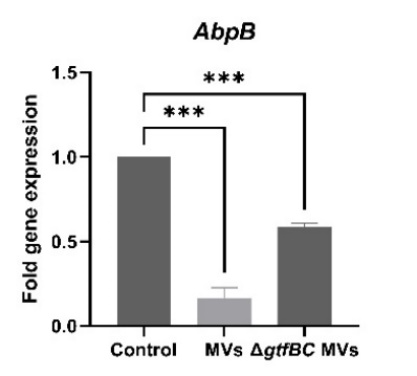** | C  **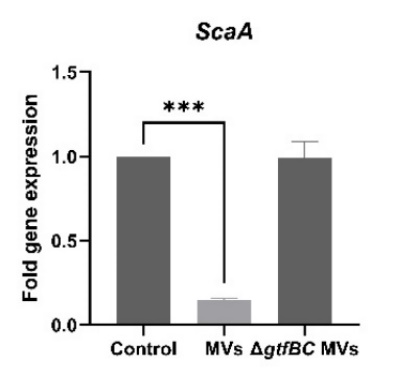** |
| --- | --- | --- |

**Figure 4. Effects of MVs on the expression levels of adhesion genes of *S. gordonii*.** The data are presented as mean ± SD from three independent experiments (n = 3, ∗∗∗*P* < 0.001).

**Table 1.** **Primers used in this study**.

| Primer name | Sequences (5’ - 3’) | Reference |
| --- | --- | --- |
| *AbpA* | Forward: TGATGCAGTTGAAGGTGGAA | (Zhang et al. 2005) |
|  | Reverse: TAGCTGCACCAACACGTTTC |  |
| *AbpB* | Forward: CAAAAACTCCGGAAAAACCA |  |
|  | Reverse: GGAGCTTGACTCGGTTCTTG |  |
| *ScaA* | Forward: CACCGAAGAAGAAGGCACTC |  |
|  | Reverse: TGTCTCCATCTTCGCCTTTT |  |

**References:**

Zhang Y, Lei Y, Nobbs A, Khammanivong A, Herzberg MC (2005) Inactivation of Streptococcus gordonii SspAB alters expression of multiple adhesin genes. Infect Immun 73(6):3351-7 doi:10.1128/IAI.73.6.3351-3357.2005
